# The Frequency and Effect of Granulocytic Myeloid-Derived Suppressor Cells on Mycobacterial Survival in Patients With Tuberculosis: A Preliminary Report

**DOI:** 10.3389/fimmu.2021.676679

**Published:** 2021-06-01

**Authors:** Malika Davids, Anil Pooran, Liezel Smith, Michele Tomasicchio, Keertan Dheda

**Affiliations:** ^1^ Centre for Lung Infection and Immunity, Division of Pulmonology, Department of Medicine and UCT Lung Institute & South African MRC/UCT Centre for the Study of Antimicrobial Resistance, University of Cape Town, Cape Town, South Africa; ^2^ DSI-NRF Centre of Excellence for Biomedical Tuberculosis Research; South African Medical Research Council Centre for Tuberculosis Research; Division of Molecular Biology and Human Genetics, Faculty of Medicine and Health Sciences, Stellenbosch University, Cape Town, South Africa; ^3^ Department of Immunology and Infection, Faculty of Infectious and Tropical Diseases, London School of Hygiene and Tropical Medicine, London, United Kingdom

**Keywords:** myeloid derived suppressor cells, MDSC, tuberculosis, immunology, biomarkers

## Abstract

**Introduction:**

Protective host responses in those exposed to or infected with tuberculosis (TB) is thought to require a delicate balance between pro-inflammatory and regulatory immune responses. Myeloid-derived suppressor cells (MDSCs), regulatory cells that dampen T-cell function, have been described in cancer and other infectious diseases but there are limited data on their role in TB.

**Methods:**

Peripheral blood was obtained from patients with active pulmonary TB and participants with presumed latent TB infection (LTBI) from Cape Town, South Africa. MDSC frequency was ascertained by flow cytometry. Purified MDSCs were used to assess (i) their suppressive effect on T-cell proliferation using a Ki67 flow cytometric assay and (ii) their effect on mycobacterial containment by co-culturing with H37*Rv*-infected monocyte-derived macrophages and autologous pre-primed effector T-cells with or without MDSCs. Mycobacterial containment was measured by plating colony forming units (CFU).

**Results:**

MDSCs (CD15^+^HLA-DR^-^CD33^+^) had significantly higher median frequencies (IQR) in patients with active TB (n=10) versus LTBI (n= 10) [8.2% (6.8-10.7) versus 42.2% (27–56) respectively; p=0.001]. Compared to MDSC-depleted peripheral blood mononuclear and effector T cell populations, dilutions of purified MDSCs isolated from active TB patients suppressed T-cell proliferation by up to 72% (n=6; p=0.03) and significantly subverted effector T-cell-mediated containment of H37*Rv* in monocyte-derived macrophages (n=7; 0.6% versus 8.5%; p=0.02).

**Conclusion:**

Collectively, these data suggest that circulating MDSCs are induced during active TB disease and can functionally suppress T-cell proliferation and subvert mycobacterial containment. These data may inform the design of vaccines and immunotherapeutic interventions against TB but further studies are required to understand the mechanisms underpinning the effects of MDSCs.

## Introduction

Tuberculosis (TB) is the leading cause of death due to a single infectious agent (1). Although TB is largely a curable disease with a global treatment success rate of ~85%, almost 1.5 million people succumbed to the disease in 2019 (1). This is likely to worsen due the rapid emergence and global spread of drug resistant forms of the disease which threaten to derail advances in TB control (2). Eradication of TB is only likely to be achieved with an effective vaccine. Currently, the only licensed TB vaccine is an attenuated strain of *Mycobacterium bovis* (*M.bv*), BCG (3). BCG confers protection against severe forms of TB disease in children but offers limited protection (~30%) against adult forms of pulmonary disease (4). Data from recently trialled vaccine candidates have not offered much hope. For instance, MVA85A did not show any improvement compared to BCG despite compelling pre-clinical animal data (5), and the more recently trailed M72/AS01 performed much better but was still only associated with an efficacy of only ~50% (6). These studies and their outcomes highlight our incomplete understanding of the immune mechanisms underpinning protective immunity and bacterial persistence.

One of the central hallmarks of active TB disease is a failed T-cell effector immune response (7). However, there is still much that is not known about the specific immune mechanisms underpinning failed T-cell immunity (8). In active TB disease immunity associated with recovery is traditionally thought to be primarily driven by pro-inflammatory effector T-cells, and cytotoxic CD8 cells (8). However, the host immunity also involves several regulatory mechanisms for suppressing T-cell responses against *M. tb*-specific antigens, which may be leveraged by pathogens to their advantage. Such strategies may also underpin the extensive immunopathology associated with TB.

Myeloid-derived suppressor cells (MDSCs) is one type of regulatory cell that potently regulates cancer immunity at the site of pathology (9). MDSCs have a morphology similar to granulocytes and/or monocytes (10). In healthy individuals, immature myeloid cells are generated in the bone marrow and rapidly differentiate into mature macrophages, dendritic cells, or granulocytes (10). MDSCs can broadly be classified into three groups: 1) early-stage-MDSC (LIN1^-^HLA-DR^-/low^ CD11B^+^CD33^+^), 2) polymorphonuclear-MDSC (HLA-DR^-/low^ CD14^-^CD15^+^CD33^+/dim^), and 3) monocytic-MDSC (HLA-DR^-/low^CD14^+^CD15^-^CD33^+^) (10). This report specifically focuses on polymorphonuclear-MDSCs (PMN-MDSCs), also known as granulocytic-MDSCs.

Others have previously shown that regulatory pathways (11, 12), including MDSCs are upregulated in patients with active TB (13–17), their frequency in peripheral blood decreases with successful treatment (14), and MDSCs are able to inhibit T-cell proliferation (13). However, data are limited to a handful of studies (less than 5), and there are hardly any data from humans or TB-endemic settings (13). Furthermore, there are no published data about the biological significance of these cells and the ability of MDSCs to directly restrict mycobacterial growth. Thus, their role during *M. tb* infection remains unclear. To address these knowledge gaps, we explored the levels of M-MDSCs in patients with active TB versus those who are latently infected, and whether they abrogated the ability of effector T-cells to contain mycobacterial growth.

## Methods

### Participants and Ethical Approval

HIV-uninfected participants were recruited from various primary healthcare clinics around Cape Town between January 2017 and February 2019. Presumed LTBI participants were healthy asymptomatic individuals with no clinical or radiological evidence of previous or active TB disease and were both tuberculin skin test (TST; induration >10mm) and interferon-gamma release assay (QuantiFERON^®^-TB Gold) positive. TB patients were microbiologically confirmed by sputum smear microscopy, Xpert MTB/RIF, and/or MGIT sputum culture, and had <1 week of anti-TB therapy at the time of recruitment. Informed written consent was obtained from all patients and the study was approved by the UCT Research Ethics Committee. Participants were excluded from the study if they were HIV-infected, pregnant or younger than 18 years of age.

### Flow Cytometry

Peripheral blood was obtained from all study participants by venipuncture. 100μl whole blood were seeded in a 96-well tissue culture plate and either left unstimulated or stimulated with 12μg/ml purified protein derivative (PPD; Statens Serum Institute) overnight at 37°C with 5% CO_2_. The cells were subsequently stained using fluorescently labelled antibodies specific for cell surface markers [CD14, CD33, HLA-DR and CD15; (BD Biosciences, Ebiosciences, Biolegend)]. Thereafter, the cells were fixed in 4% paraformaldehyde and data acquired on an LSRII flow cytometer.

### Myeloid-Derived Suppressor Cells Isolation

MDSCs were isolated as previously described by Lechner et al. (18), with modifications. Briefly, whole blood was separated over a density gradient (Histopaque 1077, Sigma-Aldrich) into the 1) plasma, 2) mononuclear cell (PBMC) layer, and 3) erythrocyte/granulocyte layer. The layer containing erythrocytes and granulocytes were harvested and erythrocytes were lysed (BD cell lysis solution). The granulocytic cell fraction was incubated with CD15 MACSiBead particles for positive selection of CD15 cells. HLA-DR^-^ cells were isolated from the CD15^+^ cell fraction by immuno-magnetic separation (MACS beads, MACS LS-column, Miltenyi Biotec). The CD15^+^HLA-DR^-^ cell fraction was incubated with CD33 microbeads for positive selection of CD15^+^HLA-DR^-^CD33^+^ cells (Miltenyi Biotec). Purity of the MDSC population exceeded 95% as confirmed by flow cytometry.

### Ki67 Suppression Assay

The suppressive effect of MDSCs on T-cell proliferation was evaluated using a Ki67 flow cytometry proliferation assay, as previously described (11). Briefly, on day 0, PBMCs were co-cultured with purified MDSCs as follows: duplicate wells containing 0.5x10^6^ PBMCs were stimulated with PPD (12μg/ml) to generate effector T-cells and co-cultured with MDSCs at ratios of 1:1, 2:1 and 4:1 (PBMCs: MDSCs) for 6 days at 37°C and 5% CO_2_. Additional experimental control wells were set up including (1) unstimulated PBMCs as a negative control; and (2) PBMCs stimulated with PPD (12μg/ml) as a proliferation control. On day 6, the cells were harvested and stained for Ki67 expression as per manufacturer instructions (Biolegend, USA). Briefly, the cells were harvested and washed with cold 70% ethanol and, after centrifugation, stained for Ki67, CD3, CD33, CD15 and HLA-DR (Biolegend, USA). Data was acquired on an LSR-II flow cytometer and analysed using FACSDiva software. The percentage (%) proliferation was calculated as follows: [(% MDSC^+^Ki67^+^ cells in experimental condition/% MDSC^+^Ki67^+^ cells in proliferation control) X 100]. The percentage suppression was calculated as 100 – (% proliferation).

### Mycobacterial Containment Assay

A mycobacterial containment assay was used, as previously described (11), to determine the effect of MDSCs on the ability of PPD-driven effector T-cells to effectively contain *M. tb* within blood monocyte-derived macrophages (MDMs). 1x10^6^/ml PBMCs were seeded into a tissue culture plate and were cultured undisturbed for 5 days to allow monocytes to adhere to the plastic and differentiate into macrophages. Thereafter, the plate was washed to remove non-adherent cells and the MDMs were infected with H37*Rv* at a multiplicity of infection (MOI) of 1:1. In parallel, purified MDSCs were co-cultured with PPD-stimulated PBMCs (12μg/ml) at specific ratios [MSDC: T_eff_ at 1:2 (5x10^4^:10X10^4^ cells); and 1:4 (2.5x10^4^:10X10^4^ cells)] and incubated for 6 days at 37°C and 5% CO_2_. The various effector T-cell/MDSC combinations were subsequently co-cultured with H37*Rv*-infected macrophages for 24 hours. Additional wells included a reference control containing H37*Rv*-infected MDMs only, a positive *M. tb* containment control containing H37*Rv*-infected MDMs co-cultured with effector T-cells (T_eff_). Intracellular *M.tb*, released by lysis of infected MDMs, was subsequently cultured on Middlebrook 7H10 agar and expressed as colony forming units per ml (CFU/ml). The percentage (%) mycobacterial containment was also reported and was defined as the reduction in *M. tb* survival compared to the reference control (H37*Rv* infected MDM only).

### Statistical Analysis

The data were tested for normality using the Shapiro-Wilk test. The Mann Whitney t test was used to assess immuno-phenotyping differences between the participant’s groups. The Wilcoxon ranked sum test was used to assess differences within participant groups. Statistical analyses were performed using GraphPad Prism version 6.0 (GraphPad software) and SPSS Statistics version 23 (SPSS Inc.).

## Results

### Clinical Characteristics of Study Participants

A total of 33 HIV-uninfected participants were recruited and classified into 2 groups (**Table 1**): microbiologically-confirmed participants with pulmonary TB (TB; n=23), and presumed-latently infected participants (LTBI; n=10) as determined by a positive tuberculin skin test (TST-positive) and Quantiferon Gold in-tube test (both positive). All participant groups were matched for age, sex and gender (**Table 1**); univariate analysis showed that good matching was achieved. All participants had no previous history of TB.

**Table 1:** Demographics and clinical characteristics of participants with latent TB infection (LTBI) and drug-sensitive TB (DS-TB).

Characteristics	Presumed-LTBI	DS-TB	All participants	p-value
**Patient numbers (n)**	10	23	33	NA
**Age (years; ^a^IQR)**	32 (28-49)	35 (26-48)	34 (26-49)	0.34
**Gender (%)**				
Male	5 (50)	12 (52)	17 (52)	0.89
Female	5 (50)	11 (48)	16 (48)	
**Ethnicity (%)**				
Black African	3 (30)	9 (39)	12 (36)	0.74
Mixed race	4 (40)	10 (44)	14 (42)	
European descent	3 (30)	4 (17)	7 (21)	
**Median duration of treatment prior to blood donation (days; ^a^IQR)**	NA	5 (4-12)	5 (4-12)	NA
**Smear status**				
Smear 1+ positive (%)	NA	3 (13)	NA	NA
Smear 2+ positive (%)	NA	12 (52)	NA	
Smear 3+ positive (%)	NA	8 (35)	NA	

NA, not applicable.

### Frequency of Myeloid-Derived Suppressor Cells in Peripheral Blood

**Figure 1:**
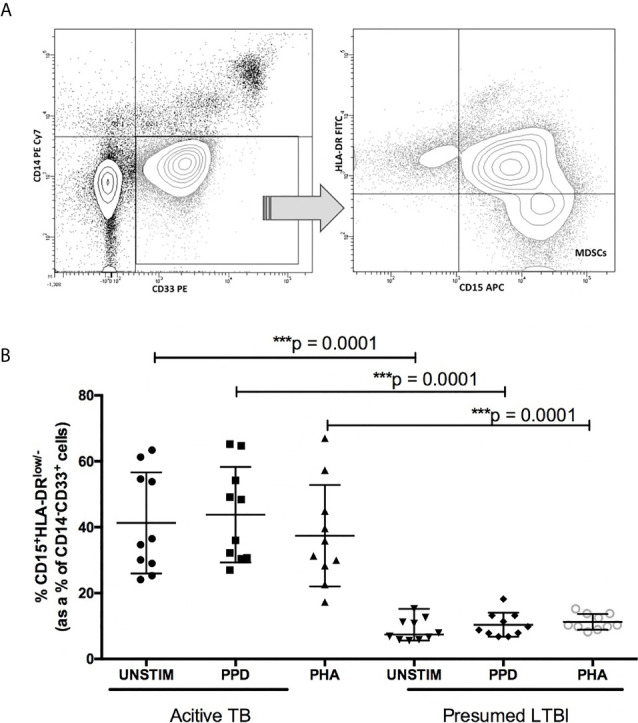
Frequency of myeloid derived suppressor cells (MDSC) in the peripheral blood compartment of participants with presumed-latent infection and active TB. **(A)** Flow cytometry gating strategy. Cells were first gated on CD14^-^CD33^+^ cells, and within this population HLA DR^low/-^CD15^+^ cells were identified. **(B)** frequency of CD14^-^CD33^+^HLA-DR^low/-^CD15^+^ MDSCs before and after stimulation with PPD and PHA. p-value of <0.05 were considered significant (Mann-Whitney unpaired t-test). ***p-value <0.0001.

In peripheral blood stimulated with PPD, we found a significant increase in the frequency of MDSCs (HLA-DR^-/low^CD14^+^CD15^-^CD33^+^) in patients with active TB (median = 42.2%; IQR: 28.1–56.3) compared to participants with LTBI (median = 8.2%; IQR: 7.6 – 10.2; p < 0.001; **Figure 1**).

### Ki67 Suppression Assay

**Figure 2:**
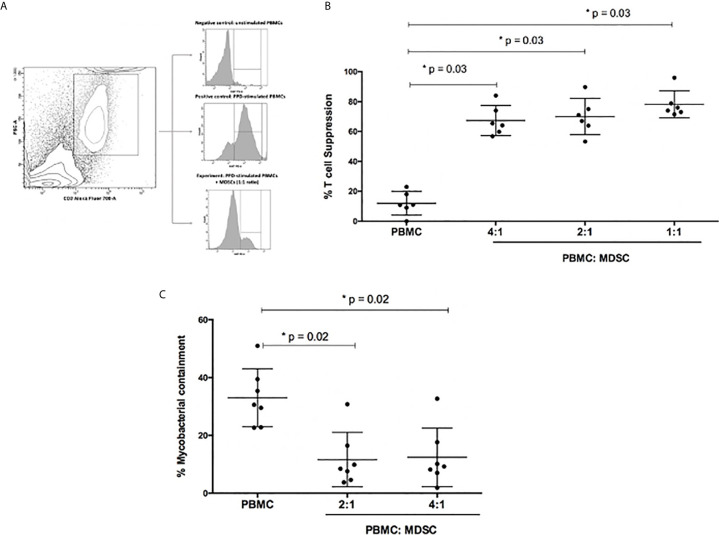
Functional capabilities of myeloid derived suppressor cells (MDSC) was tested. **(A)** Illustrates the flow cytometry gating strategy. **(B)** Outlines suppression of PPD-driven T-cell proliferation by MDSCs at different ratios. Effector T-cells were co-cultured with MDSCs at a ratio of 4:1, 2:1 and 1:1, respectively. The percentage proliferation was measured using Ki67 flow cytometry staining. **(C)** Mycobacterial containment assays using peripheral blood cells from active TB patients to determine the effect of MDSCs from TB patients on mycobacterial survival. Briefly, monocyte-derived macrophages (MDMs) were infected with H37Rv and then co-cultured with PPD pre-primed effector T-cells with or without MDSCs for 24 hours. The impact of MDSCs on mycobacterial containment was assessed by plating surviving intracellular bacteria, and the magnitude of mycobacterial containment is expressed as % of the reference control (MDM only) for each experimental condition. *p-value of <0.05 were considered significant (Wilcoxon matched pairs test).

The functional capability of the MDSC to suppress PPD-driven effector T-cell proliferative responses was evaluated using a Ki67 flow cytometry assay. MDSCs were isolated from the peripheral blood of patients with active TB disease (n=6). MDSC purity was confirmed to be greater than 95% (**Table 1**, online supplement). The proliferation control, which contained PPD-driven effector T-cells only, displayed limited T-cell suppression (median 10.95%; range: 6.75 – 19.25% T-cell suppression; **Figure 2B**). However, the addition of MDSCs to PPD-driven effector T-cells at ratios of 1:1, 2:1, and 4:1 (effector T cells: MDSCs) resulted in significant suppression of T-cells [median (range): 75% (72.62 – 95.9%), 69% (61.25 – 89.70%) and 65% (59.05 – 84%) T-cell suppression respectively; **Figure 2**].

### Mycobacterial Containment Assay

The mycobacterial containment assay was used to determine the effect of MDSC, isolated from the peripheral blood of patients with active TB (n= 7), on the ability of PPD-driven effector T-cells to effectively contain *M. tb* within autologous MDMs.

In terms of absolute CFUs, a significant decrease was observed in wells containing effector T-cells (T_eff_) co-cultured with H37*Rv*-infected MDMs (T_eff_; 4.3X10^4^ CFU/ml) compared to wells only containing H37*Rv* infected MDMs (MDM only; 7.1X10^4^ CFU/ml; p=0.0002; **Figure 2C**). However, when MDSCs and effector T-cells were co-cultured with H37*Rv*-infected MDMs, there was an increase in CFU/ml compared to the T_eff_ well (p=0.02; **Figure 2C**).

The same data, for greater clarity, were also differently expressed as % mycobacterial (*M.tb*) containment, defined as the % change in CFU/ml relative to the H37*Rv*-infected MDMs reference control. As such, the H37*Rv*-infected MDM control or reference represented 0% *M. tb* containment, or in other words, 100% *M.tb* survival. The addition of effector T-cells together with the H37*Rv*-infected MDMs, represented the “positive control”. In the positive control 30.6% *M.tb* containment was observed, however this level of containment was significantly reduced when MDSCs were introduced into the culture (2:1 = 9.2% and 4:1 = 8.5% *M.tb* containment; p=0.02).

## Discussion

We found that patients with active TB disease had significantly higher frequencies of circulating MDSCs. These cells suppressed the proliferative capabilities of PPD-specific effector T cells and attenuated mycobacterial containment *in vitro.*

The role of MDSCs in TB has been contentious. We have shown, for the first time in humans, that MDSCs can modulate T-cell activity, thus enhancing mycobacterial growth *in vitro*. Our results indicate that MDSCs can modulate an immune response that favours *M.tb* survival. The mechanism by which this occurs deserves further study. Previous murine studies that examined the direct effect of MDSCs on mycobacterial survival, showed that infected MDSCs were unable to directly kill mycobacteria (19), despite their ability to produce nitric oxide (20). Knaul et al. found that *M.tb*-infected MDSCs, similar to macrophages, can release pro-inflammatory (IL-1 and IL-6), but also anti-inflammatory cytokines (IL-10), thus providing a possible mechanism for our observations (21). However, further work in now required to study an array of possible mechanisms by which might potentially including Th2 cytokines, cell-to-cell contact, and other humoral mechanisms etc.

We found the levels of MDSC were significantly higher in patients with active TB compared to those with presumed-LTBI, and that MDSCs suppressed T-cell proliferation. Indeed, MDSCs were first described as suppressor cells recruited to tumor sites (10), where the increased levels of MDSCs significantly correlated with aggressive disease, poor patient prognosis and immune escape (18). More recently, MDSCs were shown to be upregulated during active TB disease in adults (13, 14) and in HIV-TB co-infected children (17), and in the same patients are able to suppress T cell proliferation (13, 14) and alter cytokine expression (13). However, although we confirm these observations, we have extended these findings to show that the anti-proliferative and immune-modulatory effects translate into sub-optimal mycobacterial containment.

There are several limitations to our findings. The immuno-phenotype of study participants was analysed in the peripheral blood compartment, and whether these effects occur at the site of disease remain unknown. However, harvesting cells from the lung is constrained by cost, ethical and infection control considerations. Second, our study sample size was limited, and we only recruited patients from one geographical setting. Nevertheless, we were able to demonstrate statistically significant differences between the groups, which together with the impact on proliferation, suggest that these are likely biologically meaningful observations. Third, we did not explore the immune mechanism by which MDSCs subverted the ability of T-cells to kill *M.tb*. However, this was limited by available funding but is likely to begin shortly.

Fourth, containment was used to define how MDSCs can modulate the function of effector T cells to control *M. tb.* As described in our previous publication (22), it is difficult to quantify anti-mycobacterial activity as our assay cannot distinguish between organisms that have been killed or those that have entered a non-replicating state. However, containment is a much more biologically meaningful outcome measure as compared to biomarker proxies, including cytokines or receptor signaling pathways, that may indicate protection. Although the latter are commonly used in human studies, they may merely represent a bystander effect rather than having a causal link.

In conclusion, persons with active TB disease, compared to those with presumed-LTBI, demonstrate an altered immuno-phenotype characterized by a high frequency of MDSCs. MDSCs isolated from patients with active TB disease were highly suppressive and could attenuate mycobacterial containment *in vitro.* Collectively, these data suggest a contributing role for MDSCs in the immuno-pathogenesis of TB and MDSCs may be potential targets that could be exploited to design vaccines or host-directed therapies.

## Author’s Note

LS is currently based at Stellenbosch University, but all work outlined in this study was performed when she was based at the Centre for Lung Infection and Immunity at the University of Cape Town.

## Data Availability Statement

The raw data supporting the conclusions of this article will be made available by the authors, without undue reservation.

## Ethics Statement

The studies involving human participants were reviewed and approved by University of Cape Town Research committee. Patients/participants provided their written informed consent to participate in this study.

## Author Contributions

Study concept and design: MD, AP, and KD. Laboratory work: MD, AP, LS, and MT. Analysis and interpretation: MD, AP, LS, MT, and KD. Drafting of manuscript: MD, AP, LS, MT, and KD. All authors contributed to the article and approved the submitted version.

## Funding

KD and the work presented here was supported by the South African MRC (RFA-EMU-02-2017) and the EDCTP (TMA-2015SF-1043 & TMA- 1051-TESAII).

## Supplementary Material

The Supplementary Material for this article can be found online at: https://www.frontiersin.org/articles/10.3389/fimmu.2021.676679/full#supplementary-material

## Conflict of Interest

The authors declare that the research was conducted in the absence of any commercial or financial relationships that could be construed as a potential conflict of interest.
