# Supplementary material for: The Frequency and Effect of Granulocytic Myeloid-Derived Suppressor Cells on Mycobacterial Survival in Patients With Tuberculosis: A Preliminary Report
Source: Front Immunol. 2021 Jun 1;12:676679. doi: 10.3389/fimmu.2021.676679 (PMC8207464; doi:10.3389/fimmu.2021.676679)
Supplement: Supplementary file 1 [file Table_1.pdf]

The frequency and effect of granulocytic myeloid-derived suppressor cells on mycobacterial survival in patients with tuberculosis: a preliminary report

[1]Malika Davids, [1]Anil Pooran, [1,2]Liezel Smith*, [1]Michele Tomasicchio, and [1,3]Keertan Dheda

1. Centre for Lung Infection and Immunity, Division of Pulmonology, Department of Medicine and UCT Lung Institute & South African MRC/UCT Centre for the Study of Antimicrobial Resistance, University of Cape Town, Cape Town, South Africa.
2. DSI-NRF Centre of Excellence for Biomedical Tuberculosis Research; South African Medical Research Council Centre for Tuberculosis Research; Division of Molecular Biology and Human Genetics, Faculty of Medicine and Health Sciences, Stellenbosch University, Cape Town.
3. Faculty of Infectious and Tropical Diseases, Department of Immunology and Infection, London School of Hygiene and Tropical Medicine, London, UK

Table S1: Purity of Myeloid derived suppressor cells (MDSC) isolated from active TB patients

Study participant ID	Total number of Myeloid derived suppressor cells (MDSC) isolated	Percentage MDSC purity
TB012	$1.1X10^6$ cells	97 %
TB013	$0.9X10^6$ cells	98 %
TB014	$1.6X10^6$ cells	96 %
TB015	$2.0X10^6$ cells	95 %
TB016	$1.4X10^6$ cells	98 %
TB017	$1.1X10^6$ cells	97 %
TB018	$0.8X10^6$ cells	99 %
TB019	$1.6X10^6$ cells	96 %
TB020	$1.2X10^6$ cells	96 %
TB021	$1.1X10^6$ cells	98 %
TB022	$1.8X10^6$ cells	95 %
TB023	$1.0X10^6$ cells	98 %
